# Genetic diversity and population divergences of an indigenous tree (*Coffea mauritiana*) in Reunion Island: role of climatic and geographical factors

**DOI:** 10.1038/s41437-018-0168-9

**Published:** 2018-11-26

**Authors:** Edith Garot, Thierry Joët, Marie-Christine Combes, Philippe Lashermes

**Affiliations:** grid.503155.7 IRD, University of Montpellier, DIADE, Montpellier, France

**Keywords:** Population genetics, Biodiversity

## Abstract

Oceanic islands are commonly considered as natural laboratories for studies on evolution and speciation. The evolutionary specificities of islands associated with species biology provide unique scenarios to study the role of geography and climate in driving population divergence. However, few studies have addressed this subject in small oceanic islands with heterogeneous climates. Being widely distributed in Reunion Island forest, *Coffea mauritiana* represents an interesting model case for investigating patterns of within-island differentiation at small spatial scale. In this study, we examined the genetic diversity and population divergences of *C. mauritiana* using SNP markers obtained from 323 individuals across 34 locations in Reunion Island. Using redundancy analysis, we further evaluated the contribution of geographic and climatic factors to shaping genetic divergence among populations. Genetic diversity analyses revealed that accessions clustered according to the source population, with further grouping in regional clusters. Genetic relationships among the regional clusters underlined a recent process of expansion in the form of step-by-step colonization on both sides of the island. Divergence among source populations was mostly driven by the joint effect of geographic distance and climatic heterogeneity. The pattern of isolation-by-geography was in accordance with the dispersal characteristics of the species, while isolation-by-environment was mostly explained by the heterogeneous rainfall patterns, probably associated with an asynchronous flowering among populations. These findings advance our knowledge on the patterns of genetic diversity and factors of population differentiation of species native to Reunion Island, and will also usefully guide forest management for conservation.

## Introduction

Oceanic islands are de novo islands that are usually result of volcanic activity and have never been connected to landmasses (García-Verdugo and Fay 2014). Since Darwin’s observations in the Galapagos Islands, oceanic islands have been considered as ideal systems for understanding processes of evolution and speciation. Their particularities are based on several characteristics (Emerson 2002; Losos and Ricklefs 2009). First, patterns of evolution are easier to observe and interpret in oceanic islands thanks to the restricted distribution areas of the species and the simplified biota. Second, despite their small physical dimension, oceanic islands may offer a diversity of habitats with as many possible directions for diversification and speciation. Third, provided that contacts with the neighboring lands have been limited, species have evolved in relative genetic isolation and have diverged along their own evolutionary trajectories. When volcanic activity is ongoing, it also provides a dynamic evolutionary context with the creation/suppression of biological niches. All the above characteristics have favored high levels of endemism in oceanic islands that contribute to their frequent biodiversity hotspot status (Myers et al. 2000).

Reunion Island (2512 km²) is a young island belonging to the Mascarene archipelago (~−2 Ma, Chevallier and Vatin-Perignon 1982), located in the southern Indian Ocean (55°30′E; 21°10′S), around 700 km off the coast of Madagascar. Volcanic activity and erosion have shaped a high relief (Piton des Neiges, 3071 m a.s.l.) with dramatically rugged topography, leading to notable climatic heterogeneity at small spatial scale and high habitat diversity (Strasberg et al. 2005). Compared to the surrounding islands, human impact on the native forest have been limited: around one third of the original vegetation has been preserved and shows substantial endemism, with 28% of endemism in flowering plants (CBNM 2018). Human disturbances were concentrated in lowlands, almost preserving the native forest of intermediary and high elevation. Although the insular context of Reunion Island offers an excellent framework for studying patterns and processes of evolution, in fact, few studies have focused on the subject.

Gene flows by dispersal strongly influence the genetic diversity and spatial genetic structure of plant populations. Two main patterns of gene flow are reported to be associated with population divergence: isolation-by-distance (IBD) and isolation-by-environment (IBE) (Wang et al. 2013; Sexton et al. 2014). Under IBD, genetic differentiation increases with geographic distance irrespective of environmental differences among populations. It results from reduced gene flow between distant populations and drift (Wright 1943). Hence, in a case of strict IBD, the dispersal abilities of the species and the size of the population are the main factors to take into consideration to explain differentiation (Slatkin 1993). Alternatively, the pattern of IBE is depicted by increasing genetic differentiation with environmental dissimilarity independently of geographic distance. Lower gene flow occurs among populations with different environmental features (Wang et al. 2013; Sexton et al. 2014). Several mechanisms may drive the pattern of IBE including selection and local adaptation (Nosil et al. 2009; Edelaar et al. 2012), or non-random mating due to adaptation or phenotypic plasticity (Sexton et al. 2014). In practice, geographical and environmental factors are not mutually exclusive and can jointly contribute to population divergences. Especially on oceanic islands, IBD and IBE can coexist provided that the island is not too small and present substantial habitat diversity. Reunion Island offers an ideal context to assess the role of environmental factors when geographic distances are reduced. Previous studies on endemic plants showed diverse patterns for explaining population divergence according to the species (Litrico et al. 2005; Mallet et al. 2014; Jaros et al. 2016; Blambert et al. 2016; Bourgeois et al. 2018).

The genus *Coffea* (Rubiaceae) comprises 124 species most of which are distributed in tropical Africa, Madagascar and islands in the western Indian Ocean, and when *Psilanthus* species are included, even in tropical Asia and Australia (Davis et al. 2006, 2011). *Coffea* species are perennial woody bushes or trees. With the exception of *C. arabica* (2n = 4 × = 44), all *Coffea* species are diploid (2n = 2 × = 22). Among the latter, coffee trees from the Mascarene Islands (Mauritius and Reunion Island) are of monophyletic origin (Nowak et al. 2012; Hamon et al. 2017; Kainulainen et al. 2017) and form the Mascarocoffea group. The ancestral lineage of *Coffea* is estimated to have colonized the island of Mauritius at ~1.8–2.6 Ma BP, likely from either Madagascar or Africa, through long-distance dispersal events (Nowak et al. 2014). In Mauritius, four species were characterized (*C. myrtifolia*, *C. bernardiniana*, *C. macrocarpa* and *C. mauritiana*). Several cases of natural hybridization between them were described, especially considering the crossing *C. macrocarpa* × *C. mauritiana* (Dulloo and Couturon 2009). In Reunion Island, a unique species was described as a botanical form of *C. mauritiana* (Davis et al. 2006). However, the origin of *C. mauritiana* from Reunion Island and its genetic relationships with its Mauritian counterparts remain to be elucidated. Although medicinal properties of *C. mauritiana* are well known (Giraud-Techer et al. 2016), the human use of the species has remained very marginal. During the XVIII century, cultivated *C. arabica* was introduced in Mauritius and Reunion Island. However, the possibility of gene flows from the introduced *C. arabica* species to the endemic *C. mauritiana* species can be discarded due to strong reproductive barriers such as non-overlapping flowering time (Noirot et al. 2016) and differences in ploidy levels (Mahé et al. 2007).

This study focusses on genetic diversity and population divergence of an indigenous tree (*C. mauritiana*) in Reunion Island forest. *C. mauritiana* is present as small patches of individuals widely distributed throughout the rainforest (270–1500 m a.s.l.) (Dulloo et al. 1999). Its extant habitat encompasses an altitudinal gradient of temperature and contrasted rainfall regimes. Hence, *C. mauritiana* has a wide distribution in a heterogeneous landscape. Despite the lake of genomic studies on *C. mauritiana*, the species represents an interesting model case to gain insights into the patterns and factors of differentiation within a small oceanic island. Based on previous studies on endemic species (Litrico et al. 2005; Mallet et al. 2014; Jaros et al. 2016; Blambert et al. 2016; Bourgeois et al. 2018), genetic divergences between local populations were expected in spite of the small area of the island. Among factors explaining for population divergences, the importance of environmental factors has already been highlighted in Reunion Island species (Mallet et al. 2014). Hence, an influence of the environment in population divergences may also be considered here. Using genome-wide DArTseq markers, we more specifically aim to (1) examine patterns of genetic diversity of *C. mauritiana* in Reunion Island, (2) explore the genetic connectivity between *C. mauritiana* from Reunion Island and the Mauritian coffee species, (3) investigate the role of climate and geographic distance in shaping population divergences in Reunion Island, and (4) highlight historical migration and mixture events.

## Material & methods

### Plant material and DNA extraction

**Fig. 1:**
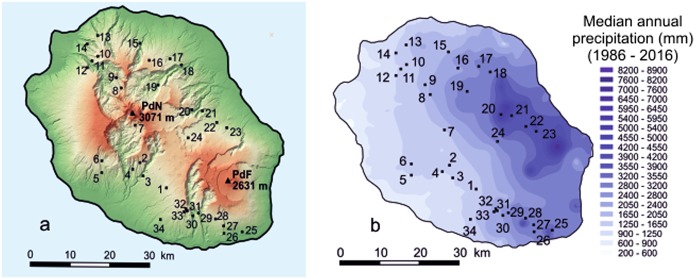
Sampling localities and climates on Reunion Island. **a** Provenance of source populations of *C. mauritiana* on Reunion Island. PdN Piton des Neiges, PdF Piton de la Fournaise. **b** Spatial rainfall distribution over Reunion Island

*C. mauritiana* is a sub-canopy tree widely distributed in the forest of low and intermediary altitude from Reunion Island. In its natural range, the species is present in diverse conditions of temperature (13–24 °C in mean annual temperature) and rainfall (from 1100 to up to 5000 mm in median annual precipitation). Apart from lowlands, the original habitat of *C. mauritiana* in Reunion Island was almost well preserved from human disturbances. Collecting missions were organized mainly based on previous records available in the “Mascarine” database (Mascarine, *système d’information sur la flore et les habitats de La Réunion*) developed by the *Conservatoire Botanique National de Mascarin* (CBNM, Saint-Leu, Reunion Island). Sampling was designed to cover the environmental range of habitats and the geographic distribution of the species in Reunion Island as well as possible. The number of samples per source population (~ 10) differed depending on accessibility and on the number of trees identified. Special care was taken to collect distantly located trees to minimize relatedness between the trees sampled. Nineteen additional *C. mauritiana* trees were sampled at the international *Coffea* collection (IRD, Saint-Pierre, Reunion Island), not only providing more individuals per source population but also individuals from two additional source populations (Salazie and Piton Fougères). In all, a total of 326 *C. mauritiana* individuals from 34 source populations were collected (Fig. 1a, Table 1). To develop phylogenetic approaches, eight representatives of the Mauritian endemic coffee species were also sampled at the IRD *Coffea* collection, as well as representatives of the main other biogeographic groups identified for *Coffea* species (West and Central Africa, East Africa and Madagascar) (Table S1).

**Table 1:**
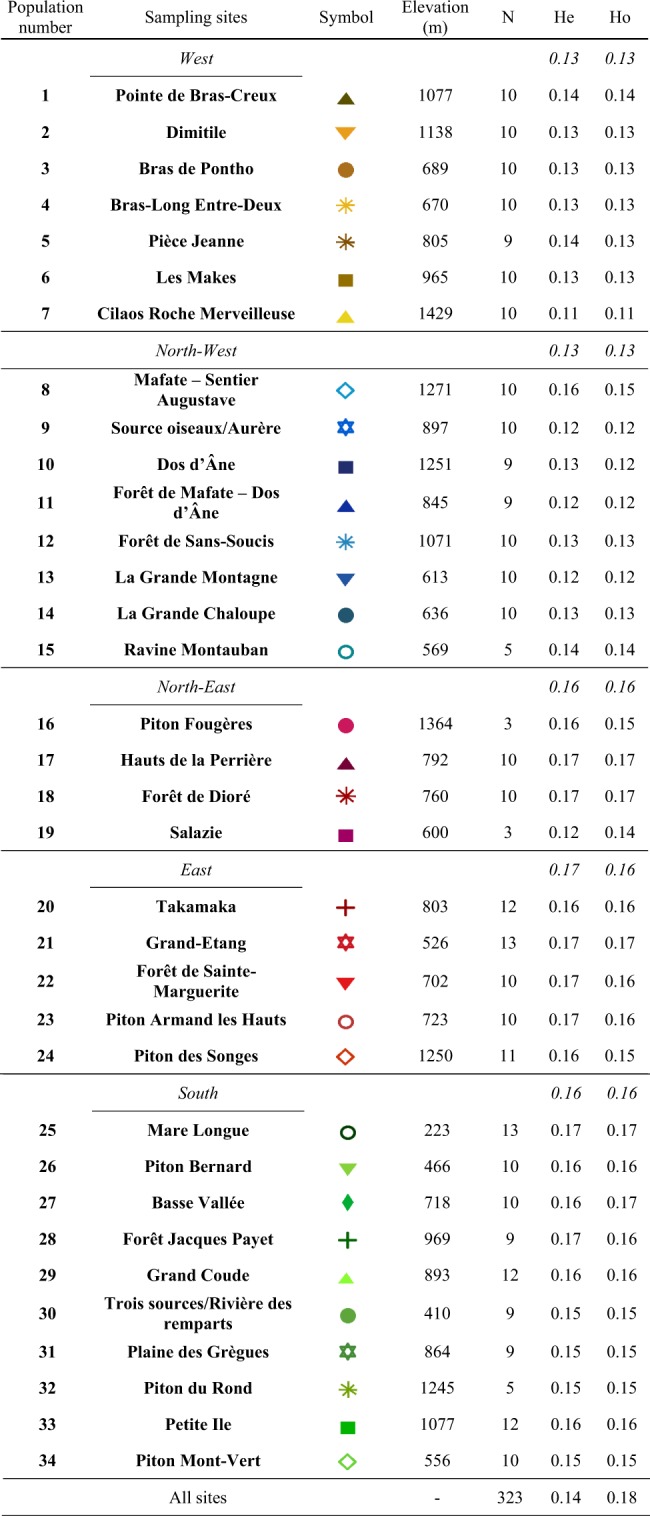
Source population characteristics and summary statistics of genetic diversity estimates obtained from the analysis of 3953 SNP markers

Arithmetic means for each region are in italics.

*N* number of individual plants used for subsequent analyses, *He* expected heterozygosity, *Ho* observed heterozygosity.

Leaves were freeze-dried and stored at −80 °C until DNA extraction. Approximately 70 mg of lyophilized leaf material were used for genomic DNA extraction using an in-house MATAB based protocol. The DNA was treated with RNase A (Promega, USA). DNA concentration was estimated by 1% (*w/v*) agarose gel electrophoresis, in comparison with a standard series of lambda *DNA*/*EcoRI* + *HindIII* markers (Promega, USA).

### Climatic data

The climate in Reunion Island is mainly characterized by a thermal gradient associated with elevation (annual mean temperature 0–25 °C) and contrasted annual rainfall regimes (500–11,000 mm; Météo-France 2018). High rainfall is recorded on the windward side of the island (east coast), whereas the climate is substantially drier on the leeward side (west coast; Fig. 1b).

For each sampled source population, daily climatic data (precipitation, temperature, potential evapotranspiration and incident radiation) were obtained from the METEOR meteorological data repository (CIRAD, UR AÏDA 2018) for the period 2002–2016 and using an optimized interpolation of records for any geographical point in Reunion Island (https://smartis.re/METEOR, Chopart et al. 2002). The number of meteorological stations used for interpolation varies from 57 (temperature records) to 124 (rainfall records) according to the climatic record being considered. Twelve derived climate variables were calculated as mean values over the period: mean of minimum, average, and maximum daily temperature (Tmin, Tmean, Tmax, respectively, °C), average daily temperature amplitude (Trange, °C), mean of minimum, average, and maximum monthly precipitation (Pmin, Pmean, Pmax, mm), average monthly potential evapotranspiration (PET, mm), mean number of consecutive dry days (CDD, counts of days with less than 5 mm of precipitation), rainfall concentration index (RCI, mm) (average intra-annual variation in monthly precipitation), drought severity index (DSI, dry period weighted intensity), average water balance of driest months (WBmin, mm). Pearson correlation coefficients and their *p*-value adjusted by Holm’s method were calculated between all complete pairs of variables using the Rcmdr R package (Fox J 2005) and a principal component analysis (PCA) was performed in R 3.3.2 to determine the main climatic factors differentiating the natural habitats.

### DArTseq analysis

Genomic DNA from each accession was standardized to a concentration of 50–100 ng μl^−1^ and sent to Diversity Array Technology Pty. Ltd. Australia (http://www.diversityarrays.com/). One DNA sample was duplicated to be used as technical replicate. Samples were genotyped by Diversity Arrays Technology Pty Ltd using the DArTseq platform (Sansaloni et al. 2011). The enzyme combination *PstI/HapII* was selected for library construction and fragments were sequenced using Illumina technology (Illumina, San Diego, CA, USA). Genotype data were generated using proprietary analytical pipelines (DArT Pty Ltd). Identical sequences were collapsed into “fastqcall files”. These files were then processed to produce “SNP” tables (codominant markers) and “silicoDArT” tables (dominant markers). Finally, the different sequences were blasted (E-value = 5×10^−7^; Minimum sequence identity = 80%) against the publicly available *C. canephora* genome (http://coffee-genome.org/) to determine the corresponding genomic positions.

### Data filtering for clustering analysis

The DArTseq-derived SNP dataset was subset for phylogenetic analysis purposes. Regarding *C. mauritiana* from Reunion Island, seven accessions were selected to cover the natural distribution of the species. Concerning the other DArTseq genotyped species, all accessions were included. The resulting dataset included 24 accessions. SNP showing missing values (not available, NAs) higher than 8% in all *Coffea* accessions were discarded. SNP with minor allele frequency (MAF) lower than 0.02 (just one alternative allele) among the seven *C. mauritiana* from Reunion Island were filtered out. Regarding the other *Coffea* accessions, no MAF filtering was performed.

The genetic distance (simple matching) between accessions was calculated from the filtered SNPs using DARwin 6.0.14 software (Perrier and Jacquemoud-Collet 2006), and a neighbor-joining tree was computed. Robustness of the nodes was evaluated by bootstrapping (1000 bootstraps).

### Data filtering for population genetic studies and assessment of genetic diversity

The DArTseq-derived SNP data set corresponding to the *C. mauritiana* samples from Reunion Island was filtered to only retain individuals with NAs < 25%. SNPs with NAs > 5% and MAF < 0.002 were discarded. The expected heterozygosity (He), and the observed heterozygosity (Ho) were calculated for the 34 source populations, using ARLEQUIN version 3.5.2 (Excoffier et al. 2005). Next, average He and Ho were calculated per geographic regions of origin (Table 1**)**.

Otherwise, the SilicoDArT data set was filtered to avoid markers with NAs >5%; markers for which a restriction site was detected only once; markers whose location on *C. canephora* genome could not be determined. Individuals were also filtered to only retain those with NAs <5%.

### Assessment of genetic diversity structure in *C. mauritiana*

A tree-based method was first performed using DARwin. Genetic distance was computed between each pair of individuals (at least 80% of pairwise completion; 1000 bootstraps) using a simple matching distance and the corresponding neighbor-joining tree was computed.

Next, the sNMF program (sparse non-negative matrix factorization algorithms) implemented in the LEA R package v. 2.0.0. (Frichot and François 2015) was used to infer individual ancestry coefficients. The program relies on sparse non-negative matrix factorization (sNMF) algorithms to estimate population clustering, and to compute least squares estimates of the ancestry coefficients (Frichot et al. 2014). sNMF algorithms were run 100 times for each putative number of ancestral populations (*K*), ranging from *K* = 1–30 (*α* = 1000). The cross-entropy criteria (CEC) helped to choose the optimal *K* value, a smaller value of the CEC generally indicating a better run (Frichot et al. 2014). The *K* values minimizing the CEC were first retained. The Q matrices and the bar plots associated with each selected *K* value were also screened to help in decision. The *K* value leading to the lowest number of admixture events and greatest genetic proximity of individuals originating from the same source population was selected. Finally, the best run (e.g. lowest CEC) out of the 100 runs corresponding to the selected *K* was retained for the graphical representation of ancestry estimates.

### Population differentiation analysis

Subsequent analyses were performed on 32 source populations, excluding source populations comprising fewer than 5 individuals. Analyses of molecular variance (AMOVA) were performed using ARLEQUIN. The tested model was the partitioning of total variance among the inferred genetic clusters from the sNMF analysis, among source populations within clusters, among individuals within source populations and within all individuals. When individuals from the same source population were distributed in several clusters, or showed more than 30% admixture, these individuals were discarded from the analysis. Weir and Cockerham’s F_ST_ was also computed among pairs of the 32 source populations retained, using ARLEQUIN. The statistical significance of the Weir and Cockerham’s F_ST_ was assessed by 1000 random permutations of individuals among populations.

### SNP filtering and Treemix analysis

The topology of relationships and migrations between the K populations of *C. mauritiana* were assessed by Treemix v. 1. 12 (Pickrell and Pritchard 2012) using population allelic frequencies estimated from the above mentioned filtered SNP dataset. As *C. myrtifolia* and *C. bernardiniana* were genotyped for three accessions respectively, these species were set as outgroups.

The DArTseq-derived SNP dataset was subset for including *C. mauritiana* from Reunion Island, *C. myrtifolia* and *C. bernardiniana* samples. The resulting dataset was filtered to discard SNPs with missing data and markers with MAF <0.002. Two different filtering methods were used to account for linkage disequilibrium, depending on whether information was available on the position on the coffee reference genome or not. When information on the position of the SNP was available, SNPs were filtered for *r*² < 0.2 using the bigsnpr and bigstatsr R package (function *snp_clumping*; Privé et al. 2018). When no information on position was available, only one randomly chosen SNP per sequence was retained.

The dataset was divided into windows of 20 SNPs to account for potential remaining linkage disequilibrium by jackknifing. Up to three migration edges were tested (*m* ranging from 1–3). Each Treemix analysis was computed three times to ensure consistency of the results. As all three runs were consistent, only one run is presented in the results.

The three-population test *f3*(X;Y,W) included in the TreeMix package was performed to test for mixture events for all combinations of three points out of the *K* clusters of *C. mauritiana* samples. A window of 20 SNPs was used to account for linkage disequilibrium by jackknifing. A negative *f3* statistic provided evidence that X originated from a mixture between Y and W. According to Reich et al. (2009), *Z*-scores below −2 were considered as significant.

### Effects of environmental and geographical factors on reproductive isolation

A distance-based redundancy analysis (dbRDA) was used to estimate the relative contribution of IBD and IBE to the differentiation of *C. mauritiana* source populations. The analysis consisted in multivariate linear regression between a first matrix of dependent variables (pairwise genetic distances) and a second matrix of explanatory variables (either geographic or environmental factors or both) where collinearity among variables must be minimized (Legendre and Legendre 1998; Orsini et al. 2013). The pairwise F_ST_ were set as dependent variable. The explanatory variables were set as follows: (i) Geographic variables. Principal coordinates of neighborhood matrix (PCNM) was used to transform the original geographical matrix of Euclidean distances into uncorrelated vectors. As positive eigenvectors better describe the geographical distance (Dray et al. 2006), we kept the eigenvalues of first four positive vectors (geo1, geo2, geo3, geo4) to account for patterns of IBD. (ii) Environmental variables. Climatic variables that minimized collinearity were selected previous to dbRDA. Taking into consideration both the results of Pearson’s correlations and PCA analyses, five climatic variables were retained: Tmean, Trange, CDD, PET, RCI (Fig. S1, Table S2).

dbRDA was performed using the function *capscale* in the vegan R package v. 2.4-2 (Oksanen et al. 2017). To identify the best model, the dbRDA was run on nested models, which optimizes the model by taking the Akaike information criterion (AIC) into consideration. Both forward and backward processes were used to select direction. The significance of the joint effect of predictors as well as their marginal effects were tested using multivariate F-statistics with 10,000 permutations. Multi-collinearity among the explanatory variables was also evaluated by computing the variance inflation factor (VIF), a VIF <10 being indicative of low multi-collinearity in the variables. The R²_adj_ was computed to evaluate the quality of the model. This procedure was implemented for the 32 source populations which pairwise F_ST_ was available, and was next implemented for 10 source populations from the southern part of the island. The contribution of each variable from the optimized model, kept independently from the remaining explanatory variables, was estimated by partial dbRDA. It also enabled estimation of IBD (sum of contributions of variables of geography) and IBE (sum of contributions of environmental variables). Finally, the relative contribution of both IBD and IBE and their intersection was estimated by variance partitioning.

## Results

### DArTseq analysis and SNP detection

A total of 31,398 codominant SNPs and 16,775 SilicoDArTs were obtained from the DArTseq analysis of the overall coffee accessions. In the analysis, special attention was paid to the SNP data. The average genotype call rate was of 83.2%, with SNP scoring reproducibility of 99.7%. The rate of SNP genotyping error was 1.7%.

### Origin of *C. mauritiana* from Reunion Island and genetic connectivity between *Mascarocoffea* species

**Fig. 2:**
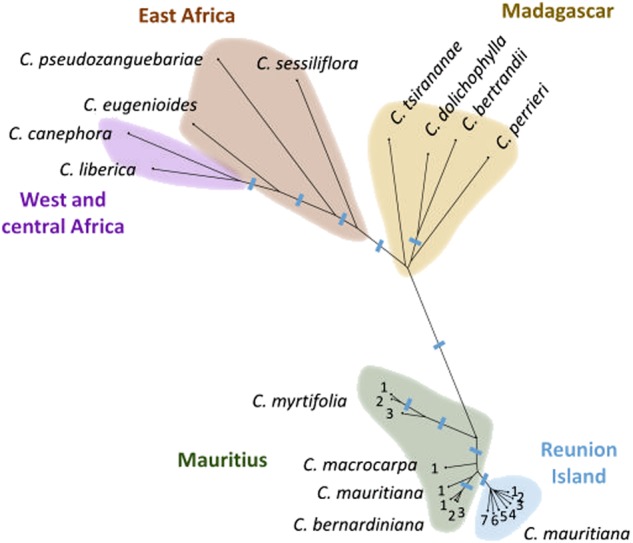
Unweighted NJ tree based on simple matching distances estimated from 2251 SNP markers, among 24 coffee accessions. Clades supported by a bootstrap value of 100% are indicated by a blue dash

Genetic distances among the 24 selected coffee accessions were calculated from the 2251 filtered SNPs. The resulting neighbor-joining tree showed that accessions were grouped according to their geographic origin (Fig. 2). Among the Mauritian representatives, *C. myrtifolia* appeared to be the most divergent, while *C. macrocarpa*, *C. mauritiana*, and *C. bernardiniana* were more closely related. Accessions of *C. mauritiana* from Reunion Island formed a distinct cluster that is divergent from the Mauritian accessions. This cluster was supported by high bootstrap values. Finally, *C. mauritiana* from Reunion Island was as distant from *C. bernardiana* representatives, *C. macrocarpa* and *C. mauritiana* from Mauritius.

### SNP filtering and assessment of genetic diversity

The complete DArTseq-derived SNP dataset was filtered for quality criteria leading to 3953 SNPs from 323 *C. mauritiana* samples from Reunion Island. This dataset was considered for analysis of genetic diversity and population structure. Total expected heterozygosity (He) and observed heterozygosity (Ho) among the 34 *C. mauritiana* source populations collected in Reunion Island were 0.14 and 0.18, respectively (Table 1). He values were low among source populations from the north-western and western parts of the island (He values <0.15), except for source population 8 (He = 0.16). Source populations from the east (mean He = 0.17), north–east (mean He = 0.16) and south (mean He = 0.16) showed the highest He values. The same pattern was observed regarding Ho values.

### Detailed assessment of population genetic structure

**Fig. 3:**
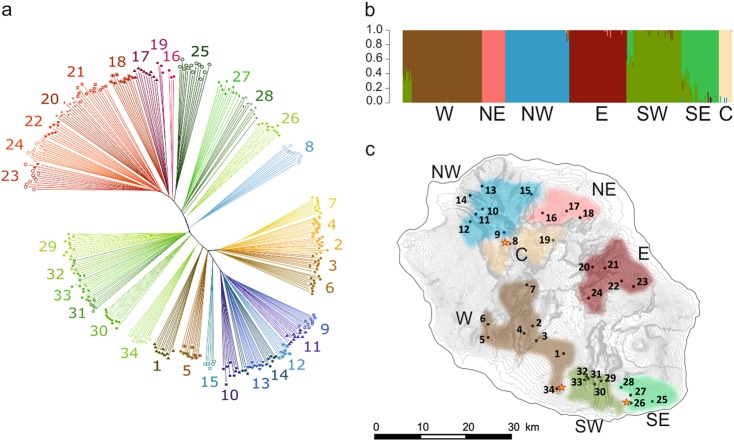
Spatial genetic structure of 323 *C. mauritiana* trees across 34 sampling sites in Reunion Island, estimated from 3953 SNP markers. **a** Unweighted NJ tree based on simple matching distances. Correspondence between the population number and the name of the population are listed in Table 1. Colors distinguish the main geographical sampling regions. Red: east and north-east; Blue: north-west; Yellow and brown: west; Green: south. **b** Group assignment from sNMF at *K* = 7. Biogeographic regions associated with the clusters are represented by seven colors. Each individual tree is represented by a bar and coefficient ancestry relative to each cluster is indicated by colored segments. W western genetic group, NE north-eastern genetic group, NW north-western genetic group, E eastern genetic group, SW south-western genetic group, SE south-eastern genetic group, C central genetic group. **c** Spatial representation of the genetic structure of *C. mauritiana* populations obtained from sNMF analysis (*K* = 7). Each sampling site is represented by a dot. Colors distinguish the genetic groups inferred from sNMF. Yellow stars indicate source populations showing admixed individuals

The overall structure in the panel was visualized in a neighbor-joining (NJ) tree (Fig. 3a). Individuals belonging to the same source population preferentially clustered together, leading to a clear separation between most source populations. The majority of bootstrap values associated with clusters of individuals from the same source population were higher than 80%. Geographically close source populations also tended to cluster together, indicating genetic proximity according to geographic distribution. This mostly concerned north-eastern and eastern (17, 18, 20, 21, 22, 23, 24), north-western (9, 10, 11, 12, 13, 13, 14), western (2, 3, 4, 5, 6), and southern regions (south-eastern: 27, 28; south-western: 29, 31, 32, 33). It should be noted that within the eastern, north-eastern, western, and southern regions, some individuals were not grouped according to the source populations. Furthermore, the shortest orthodromic distances were recorded between source populations in the southern region (32–33: 870 m; 31–33: 840 m; 31–32: 615 m) (Table S3).

The overall observation of individual clustering according to source population affiliation was confirmed by the NJ tree using filtered SilicoDArT markers (Fig. S2). The tendency of geographically close populations to be grouped together was also observed. As reported previously, individuals from the western and south-western regions displayed imperfect grouping according to the source population. In the western region, both NJ tree based on SilicoDArT markers and SNP markers showed that one individual sampled in location 3 clustered with samples from location 5 while the distance between the two locations was 10.5 km (Table S3). In contrast, eastern individuals were better grouped than previously.

The sNMF program was used to investigate population clustering of the 323 *C. mauritiana* individuals in K clusters and to infer individual ancestry coefficients. The K values ranging from 5 to 8 minimized the cross-entropy criteria (CEC) (Fig. S3a). An overview of the Q matrices (data not shown) and their graphical representations (Fig. S3b,c,d) led us to select a structure in seven main clusters as the most relevant since *K* = 7 minimized admixture events while mostly preserving the source population affiliation (Fig. 3b). The bar plot revealed that clusters of individuals matched their geographic region of origin on the island (north–east, east, west, north–west). Source populations from the south were distributed in two clusters corresponding to south–eastern and south–western regions, respectively. Moreover, one cluster (hereafter called central cluster) was formed by two source populations (8 and 19) located respectively in two neighboring cirques in the center of the island (Fig. 3c). Individuals from location 8 showed little admixture (<10%) with the north–western cluster. Individuals from the east and north–east were divided into two separate clusters. The limits of the regional clusters almost preserved the population source origin except for location 26: six individuals belonged to the south–western cluster while the other four individuals were placed in the south–eastern cluster, revealing very high admixture levels (up to 48%). Individuals from location 34 (western cluster) also showed high admixture levels (from 29 to 47%) indicating that admixture occurred between the western and south–western clusters. The results of the NJ trees, and sNMF analyses were largely consistent with an overall genetic structure at regional scale and finer substructure in source populations, except for accessions of the south–west regional cluster for which less grouping by source population was observed.

### Population differentiation analysis

Pairwise F_ST_ values were computed among the source populations comprising more than five individuals (e.g. 32 source populations). The *F*_ST_ values ranged from 0.014–0.430, with an average genetic differentiation of 0.168 (Table S4). All F_ST_ values were statistically significant (*P* < 0.001). The F_ST_ values for source populations belonging to the same regional cluster were low whereas higher F_ST_ values were observed between source populations from different regional clusters. Pairwise F_ST_ was also computed between the seven regional clusters (Table 2). Low F_ST_ values were observed between regional clusters in the east (north–east, east, and south–east) and west (north–west, west, and south–west), respectively. The highest differentiation was observed between clusters from the east and west coast, the maximum *F*_ST_ value was recorded for the north–western and eastern pair (*F*_ST_ = 0.26), while the minimum *F*_ST_ value was recorded for the north–eastern and eastern pair (*F*_ST_ = 0.04). A low F_ST_ value was also observed between southern clusters (*F*_ST_ = 0.08)

**Table 2:**
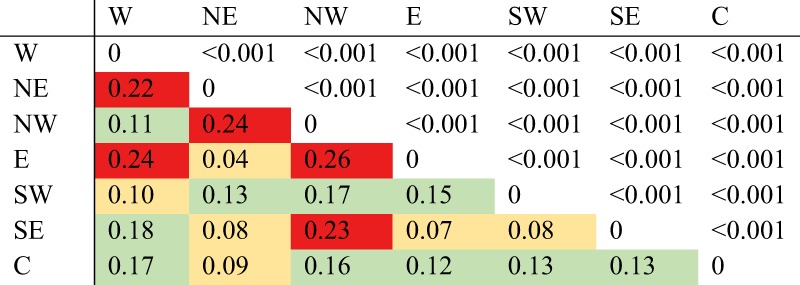
Weir and Cockerham’s pairwise *F*_ST_ calculated between the seven clusters inferred by sNMF analysis.

*F*_ST_ values are below the diagonal and *p*-values above. Yellow: *F*_ST_ ≤ 0.10; Green: 0.10 < *F*_ST_ ≤ 0.20; Red: *F*_ST_ > 0.20.

*W* western genetic group, *NE* north-eastern genetic group, *NW* north-western genetic group, *E* eastern genetic group, *SW* south-western genetic group, *SE* south-eastern genetic group, *C* central genetic group.

The results from the AMOVA were consistent with the differentiation depicted by the pairwise *F*_ST_, with 15.2% of the global variation between regional clusters, and 5.0% between source populations belonging to the same genetic cluster.

### Population genetic relationships

**Fig. 4:**
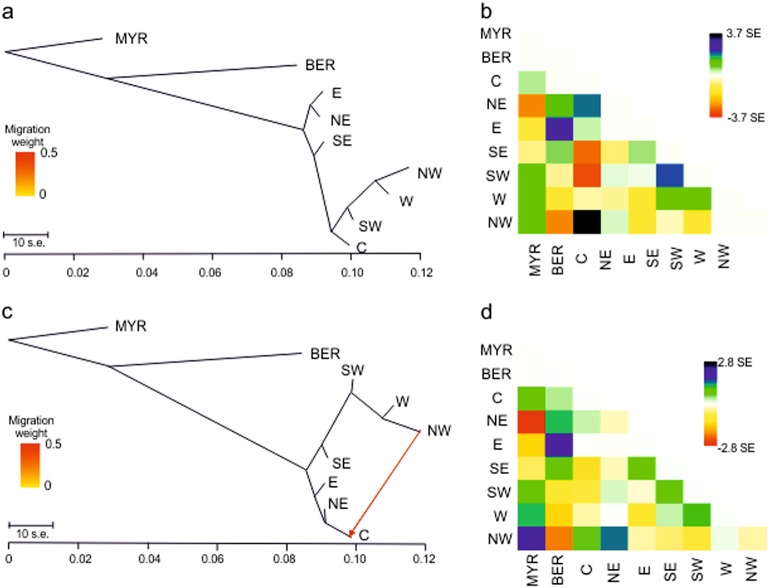
Results of TreeMix analysis of the phylogenic relationships among the seven clusters defined by sNMF analysis. *C. myrtifolia* (MYR) and *C. bernardiniana* (BER) served as outgroup populations. E eastern cluster, NE north-eastern cluster, SE south-eastern cluster, C central cluster, SW south-western cluster, W western cluster, NW north-western cluster. **a** Maximum-likelihood tree of 323 *C. mauritiana* divided into the seven population groups included in the analysis. Horizontal branch lengths are proportional to the amount of genetic drift that has occurred in each branch. **b** Residual heatmap from the tree in **a**. Pairs with high residual values are candidates for migration events. **c** Maximum- likelihood tree that best fits the data and its associated residual heatmap **d**. The migration weight indicates the proportion of ancestry deriving from the migration edge

After filtering for quality criteria and linkage disequilibrium, a total of 1280 SNPs for the 323 *C. mauritiana*, the three *C. myrtifolia* and the three *C. bernardiniana* samples were retained. A Treemix analysis was conducted to examine the topology of relationships and migration history among the seven regional clusters. With *C. myrtifolia* and *C. bernardiniana* individuals used as outgroups, the ML tree showed a split between regional clusters from the east coast (north–eastern and eastern clusters) and the other clusters from the south, the center and the west coast (Fig. 4a). The drift parameter ranged from 0.09 to 0.12, indicating low genetic divergence among the *C. mauritiana* regional clusters. The north–western cluster showed the highest drift parameter (~0.12), indicating the highest divergence from a set of ancestral allele frequencies. The tree representation captured the total variation (98.9%) well. The residual heatmap revealed that the north–western and the central clusters were the worst represented, with a standard error of 3.7 (Fig. 4b). Migration edges (parameter m) were then computed to see whether gene flow events would improve the percentage of total variance and reduce the global standard error. An optimized model was obtained by inferring one migration edge from the north–western cluster to the center (Fig. 4c). This historical migration was in accordance with the few admixture rates found in the sNMF analysis (<10%) between individuals from source population 8 (central cluster) and the north–western cluster. The ML tree supported an additional 0.54% of total variance while decreasing the global standard error and improving the representation of both the north–western and central clusters (Fig. 4d). Adding more migration edges did not significantly improve the ML tree. So an ML tree with only one migration edge was retained (weight: 41.6%; se: 0.021; *P*-value <2.23E^−6^).

An exhaustive *f3-statistics* test (three-population test) was applied to combinations of three regional groups to further investigate their mixture history. The results presented in Table S5 suggested that the south–eastern cluster originated from a mixture between the western and eastern clusters (*Z*-score: −2.12). In addition, a complex history was obtained for the south-western cluster: significant evidence was found for a mixture between western and eastern clusters (*Z*-score: −2.98), and between western and south-eastern clusters (*Z*-score: −3.29). Weak evidence was also found for a mixed origin of the central cluster from the north-eastern and the north-western clusters (*Z*-score: −1.95).

### Importance of environmental and geographical factors in explaining genetic differentiation

A series of full and partial redundancy analyses (RDA) was used to assess the amount of genetic divergence among source populations (pairwise F_ST_) explained by uncorrelated geographic vectors and climatic factors. The optimized model (AIC: −72.96; *P*-value < 0.001; *R*²_adj_: 85.0%) constrained 88.4% of total inertia, and retained seven variables: the four vectors of geographical distance, CDD (mean number of consecutive dry days), PET (average monthly potential evapotranspiration) and Tmean (mean of average daily temperature) (Table 3, Fig. S4). The VIF values were low, revealing relatively low collinearity in predictors, except for CDD (10.25). This relatively high value could be due to the strong collinearity between the geographic position and the rainfall regime in Reunion Island. The pure contribution of each independent variable was obtained after removing the confounding effects caused by the other significant factors (partial dbRDA). The contribution of geographic variables (IBD) to genetic divergence was higher than the contribution of environmental variables (IBE) (21.37 and 7.20%, respectively).

**Table 3:** Detailed results of dbRDA significance tests for the global dbRDA analysis (after removing non-significant terms) and the marginal test, considering 32 source populations of *C. mauritiana*

	dbRDA			Partial dbRDA		
	% of variance explained	d.f.	P-value	% of variance explained	d.f.	P-value
*Global analysis*	85.03	7	<0.001			
Residuals	14.97	24	–		–	–
*Marginal test*						
Geography	47.56			21.37		
geo1	22.30	1	<0.001	10.54	1	<0.001
geo2	15.24	1	<0.001	7.01	1	<0.001
geo3	6.21	1	0.006	2.51	1	0.002
geo4	3.81	1	0.048	1.31	1	0.013
Environment	23.66			7.20^a^		
CDD	9.75	1	<0.001	4.27	1	<0.001
Tmean	6.06	1	0.008	2.43	1	0.014
PET	7.85	1	0.002	3.32	1	0.004
Residual	28.76	24	–	28.76	24	–

*CDD* mean number of consecutive dry days, *Tmean* mean average daily temperature, *PET* average monthly potential evapotranspiration.

In the dbRDA analysis, the marginal effects of each constraining factor were tested through 10,000 permutation tests, by removing each term one by one from the model containing all other terms. In partial dbRDA, the pure contribution of each factor was obtained after removing the confounding effects from the other constraining factors. geo1, geo2, geo3, geo4 are uncorrelated geographic factors

^a^Due to remnant correlations between environmental variables, the estimated contribution of environmental factors in partial dbRDA (7.20%) does not correspond to the sum of individual contributions of each environmental factor (10.02%).

**Fig. 5:**
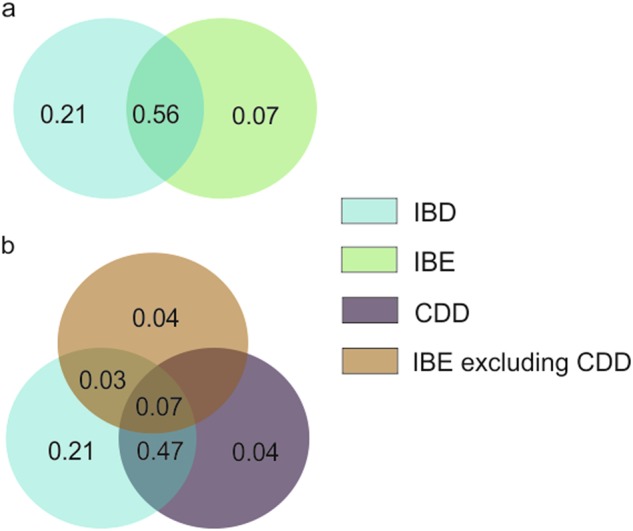
Variance partitioning results of dbRDA analyses. IBD comprises four geographic vectors (geo1, geo2, geo3, geo4). IBE comprises three environmental variables: Tmean mean of average daily temperature, CDD mean number of consecutive dry days, PET average monthly potential evapotranspiration. **a** Variance partitioning between IBD and IBE. The overlapping zone represents the intersection between IBD and IBE (IBD ∩ IBE). **b** Variance partitioning between IBD, IBE excluding CDD and CDD. Values below zero are not shown

A first variance partitioning test was then conducted to assess the relative contribution of IBD, IBE and their intersection in explaining the genetic differentiation. The largest contributor to the genetic divergence was the intersection IBD ∩ IBE explaining 56.4% (Fig. 5a). A second variance partitioning test was then performed to determine which environmental variable was mainly involved in IBD ∩ IBE (Fig. 5b). The intersection IBD ∩ CDD was responsible for 47% of variation, indicating that the main environmental factor involved in the intersection with geographic distance was CDD.

Even if we attempted to control for collinearity in data processing, this result highlighted the difficulty of disentangling the effect of geography and climate in Reunion Island, since geographically distant sites also tend to differ environmentally. To test for climatic effects while controlling for geographic distance, a dbRDA analysis was again performed on a smaller region of the island. The southern region was chosen as it contained sufficient source populations (10) to perform the analysis, with different cluster affiliation and a context of climatic heterogeneity at small spatial scale. Among the five climatic variables and the five geographic vectors, the optimized model only retained CDD and geo2. As the marginal effect of geo2 was not significant in the ANOVA table, it was removed from the explanatory variables, leading to a model with a single explanatory variable (CDD) that explained 49.4% of overall genetic differentiation (AIC: −37.92, *P*-value = 0.002, *R*²adj: 49.43%). These results confirmed the role of climate in explaining genetic divergence at reduced spatial scale and more precisely, the influence of the rainfall pattern.

## Discussion

### Colonization of Reunion Island and genetic connectivity with the Mauritian coffee species

The origin of *C. mauritiana* in Reunion Island and its genetic connectivity with the Mauritian counterparts are important to consider for a right understanding of the patterns of genetic differentiation of the species. The SNP-based clustering of *Coffea* species accessions was consistent with the known phylogeny among the *Coffea* genus (Nowak et al. 2012; Hamon et al. 2017; Kainulainen et al. 2017), supporting an independent grouping of the Mascarocoffea species. Since Mauritius is older (~−8 Ma, Moore et al. 2011) than Reunion Island (~−2 Ma, Chevallier and Vatin-Perignon 1982), an arrival of *C. mauritiana* in provenance of Mauritius was proposed as very likely (Nowak et al. 2014). Moreover considering our results, *C. mauritiana* from Reunion Island was as distant from *C. mauritiana* (Mauritius), than from *C. bernardiniana* representatives (Mauritius) and *C. macrocarpa* (Mauritius). Thus, the ancestors of Reunionnese *C. mauritiana* most likely diverged from the ancestral lineage at the origin of Mauritian *C. mauritiana*, *C. bernardiniana* and *C. macrocarpa* species. Otherwise, the cluster formed by *C. mauritiana* accessions from Reunion Island was clearly divergent from the Mauritian species, arguing for genetic isolation after the ancestral events of colonization. So, Reunion Island colonization would result from few ancient events of long distance dispersal from Mauritius.

Besides their genetic divergences, the two forms of *C. mauritiana* in Reunion Island and Mauritius were also reported to be distinct for fruits and leaves phenotypes (Leroy 1963) making doubtful the unity of the species among islands. It pointed that the biological systematics of the Mascarocoffea species would require further clarification, especially regarding the two forms of *C. mauritiana*.

### Genetic diversity

For many years, genetic variation has been considered to be reduced in plant species endemic to oceanic islands compared to their continental counterparts. This pattern was frequently attributed to the initial founder effect of colonization, limited immigration from mainland source areas and the consequence of genetic drift within small populations (Barrett et al. 1996; Frankham 1996, 1997). To better understand the level of genetic diversity in *C. mauritiana* (Ho = 0.18 and He = 0.14), indices can be compared to those obtained when considering a continental coffee species. Since *C. canephora* is an allogamous species and also presents the widest geographic distribution among *Coffea* species in Africa, it was chosen for comparison. Bearing *C. canephora* evolutionary history in mind (Cubry et al. 2013), one would thus expect much higher levels of genetic diversity in *C. canephora* than in *C. mauritiana*. Surprisingly, compared to *C. canephora*, genetic diversity indices in *C. mauritiana* were not so restricted as its evolutionary context would lead one to suppose (Ho: 0.1381 and He: 0.1763 in *C. mauritiana* versus Ho: 0.1405 and He: 0.1933 in *C. canephora*, Garavito et al. 2016). In fact, recent studies argue that after many generations on the island, genetic evidence of the initial founder effect is often no longer detected (García-Verdugo et al. 2015) and has no lasting impact on molecular evolution in endemic plants (James et al. 2016). For an appropriate interpretation of the level of genetic diversity in *C. mauritiana*, the biological characteristics of the species, such as their breeding system, aptitudes for dispersion and perenniality must be taken into account (García-Verdugo et al. 2014; Stuessy et al. 2014), as well as the island characteristics (Stuessy et al. 2014).

First, the breeding system has been shown to be a major determinant of plant genetic diversity (Glemin et al. 2006). In the *Coffea* genus, an S-RNase system was reported to be responsible for gametophytic self-incompatibility (Lashermes et al. 1996; Nowak et al. 2011; Asquini et al. 2011). Evidence for self-incompatibility in Mauritian coffee species has also been provided (Nowak et al. 2014), so self-incompatibility in *C. mauritiana* from Reunion Island is highly reliable, implying a strictly allogamous reproductive regime. Allogamy must have played a role in the persistence of significant levels of genetic diversity in *C. mauritiana*, despite a major contraction in the size of the population during its establishment on the island.

The life cycle characteristics of *C. mauritiana* could also have facilitated spatial expansion on Reunion Island. *C. mauritiana* is a woody species with a juvenile phase that can persist for several years before reproduction. This lengthy juvenile phase must have facilitated expansion and limited founder effects: delayed reproduction allowed the accumulation of several founders from the source population before reproduction began (Austerlitz et al. 2000; Born et al. 2008). Being long-lived, one individual can also contribute to the next generation several times.

Finally, geomorphological events may interfere with the interpretation of the current levels of genetic diversity. Nevertheless, its influence seems marginal here due to the age of the major geological events in Reunion Island: the main explosive period of the former volcano (Piton des Neiges) is quite ancient (around –150 ka, Gillot and Nativel 1982; Fretzdorff et al. 2000) and the current eruptions and collapses of the second volcano (Piton de la Fournaise) are restricted to a few zones in the south of the island (Michon and Saint-Ange 2008).

### A genetic structure reflecting low dispersal and environmental influence

The fine scale genetic structure found among samples provides insights into the dispersal pattern of *C. mauritiana*. Individuals clustered according to their source population affiliation, with little evidence for gene flows between source populations from the same region. *F*_ST_ values were variable (*F*_ST_ between source populations ranged from 0.01 to 0.47) revealing higher differentiation between distant source populations. These elements suggest a pattern of local dispersal with isolation-by-distance (IBD). Indeed, IBD was of considerable importance in explaining divergence between source populations (21.4% of genetic differentiation was explained by geographic distance). Pollen and seed dispersal are major determinants of spatial genetic structure. Compared to pollen transport, seed dissemination appears as marginal in several species of coffee trees. Berries seem mainly dispersed by gravity to a few meters from the mother tree and possibly transported by run-off water (Berthaud 1986). Regarding dispersal by pollen transport, in *Coffea* species it is mainly ensured by both wind and bees, but the ratio “wind-pollination” to “bee-pollination” varies considerably among species (Klein et al. 2003; Ngo et al. 2011; Noirot et al. 2016). Since the pollen of self-incompatible coffee trees like *C. mauritiana* is light and dry (Ngo et al. 2011), anemophilous dispersal is probably involved. Nevertheless, in several coffee species, the efficiency of pollen transport by wind appeared to be limited to a distance of ~100 m from the mother tree (Charrier 1972; Berthaud 1986; Ngo et al. 2011). Regarding bee-pollination, the indigenous bee (*A. m. mellifera*, Techer et al. 2017) can be an historical pollinator of *C. mauritiana*.

Long distance dispersal can occasionally occur, as exemplified in the western region: one individual clustered with the accessions of another source population sampled 10.5 km away. A few candidates can be proposed, as they are known to be efficient dispersers of several small fleshy fruits in native species: the endemic bulbul (*Hypsipetes borbonicus*, Strasberg 1996; Thébaud and Strasberg 1999), the extinct starling (*Fregilupus varius*, Cheke and Hume 2008), and an endemic bat from Reunion Island and Mauritius (*Pteropus niger*, Nyhagen et al. 2005).

Additionally, the results of our study indicate that differentiation due to limited dispersal abilities was complemented by isolation due to environmental factors (IBE, 7.2% of genetic differentiation). Whether considering all source populations or focusing on the southern ones, the indices for dry period duration, CDD, appeared as the main climatic factor associated with differentiation. In coffee species, floral bud morphogenesis is induced by a dryness period and synchronized flowering occurs at the first rain showers of the rainy season (DaMatta 2018). Because of the heterogeneous rainfall pattern in Reunion Island, the duration of drought periods and the timing of the first rain showers must differ between sites. Thus, an asynchronous flowering in *C. mauritiana* should contribute to the establishment of a reproductive barrier between source populations.

### History of population expansion

Results on relationships among the regional clusters provided further insights into present genetic diversity in *C. mauritiana* and population admixture. Overall, the low level of divergence between clusters suggests recent differentiation. The architecture of the TreeMix-derived ML tree led us to hypothesize distinct evolutionary pathways for regional groups from the eastern and western coasts, which is also supported by higher differentiation in pairwise *F*_ST_. The central montane barrier must have been a major barrier to gene flow, separating the two sides of the island (east coast versus west coast). Moreover, results suggest the recent expansion of the species through a step-by-step colonization on both sides of the island. Along the western coast, the colonization front apparently proceeded from south to north. The north–western cluster appeared to be the most differentiated (higher drift parameter) and the lowest levels of genetic diversity were also found in this regional group. Apart from separate evolution on the two sides of the island, a few ancestral mixtures were identified in the south–western and south–eastern clusters, attesting to a more complex history of population establishment in the southern part of the island. Due to proximity to the younger volcano (Piton de la Fournaise), the establishment of *C. mauritiana* in the south may have been locally impacted by several geologic events linked with volcanic activity (Michon and Saint-Ange 2008). Other gene flows between the north–western and the central clusters attest to the colonization of the central cirques by neighboring populations.

In conclusion, our study provides a comprehensive picture of the genetic diversity and structure of *C. mauritiana*, a widely represented forest tree in Reunion Island. We highlighted the joint contribution of geographic distance and rainfall regimes in shaping genetic differentiation in a small oceanic island. These results can be used to guide forest management for conservation while simultaneously favoring forest resilience to climate change (Fady et al. 2016). Moreover, the distribution of *C. mauritiana* in highly contrasted environments suggests local adaptation. Consequently, this work can also be used as a starting point for investigation of potential genetic variants underlying the adaptive traits in *C. mauritiana* populations. Finally, the information acquired on genetic relationships between populations provides insights into past forest dynamics. In depth studies are now needed to confirm the expansion scenario. Major forest transitions covering a recent period (<40,000 years before present) have been reported in Mauritius in association with climate change (Van Der Plas et al. 2012; de Boer et al. 2013). Similar reassortments in taxonomic composition of Reunion Island forest can be hypothesized.

### Data archiving

DArTseq genotype data available from the Dryad Digital Repository: 10.5061/dryad.4vc0rg0

## Electronic supplementary material

Supplementary data
