# Supplementary material for: Genetic diversity and population divergences of an indigenous tree (Coffea mauritiana) in Reunion Island: role of climatic and geographical factors
Source: Heredity (Edinb). 2018 Nov 26;122(6):833–47. doi: 10.1038/s41437-018-0168-9 (PMC6781115; doi:10.1038/s41437-018-0168-9)
Supplement: Supplementary file 1 — Supplementary data [file 41437_2018_168_MOESM1_ESM.pdf]

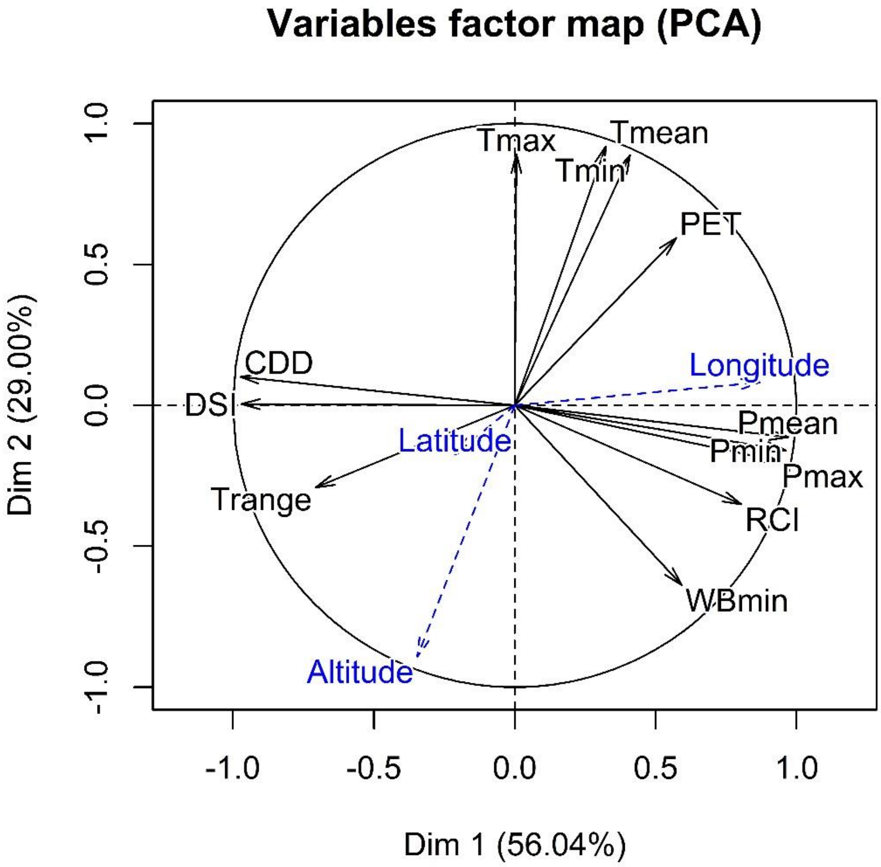

Figure S1. PCA results for the 12 climatic variables. Longitude, Latitude and Altitude were set as illustrative factors. Percentage of explained variance of each axis is given in parentheses.

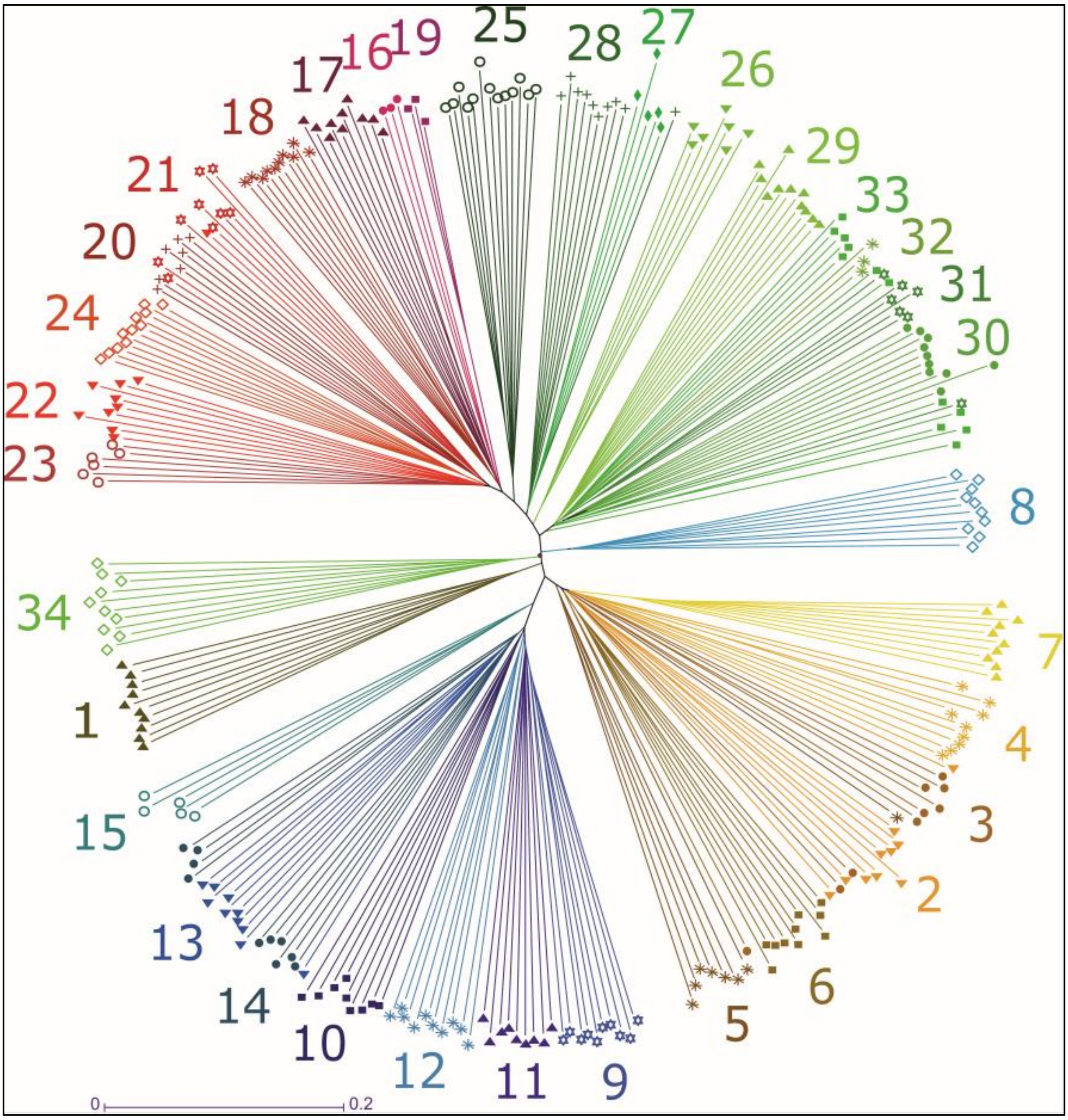

Figure S2. Unweighted NJ tree based on Jaccard's distances estimated from 1860 filtered SilicoDArT markers among 296 *C. mauritiana* (individuals < 5% NA) samples originating from 34 source populations in Reunion Island. Source population codes are given in Table 1. Colors distinguish the main geographical regions of sampling.

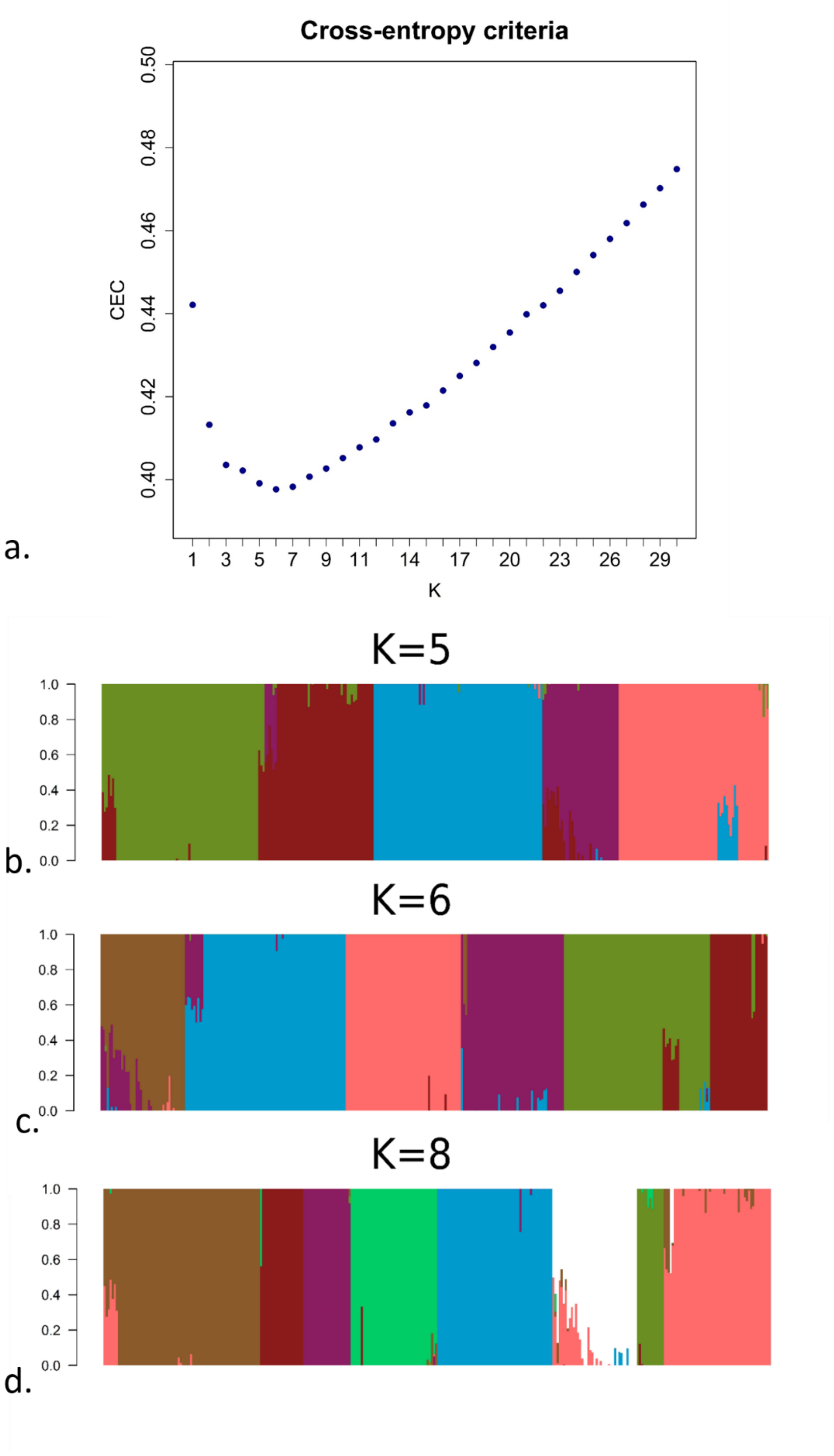

Figure S3. Inference of spatial genetic structure using sNMF. **(a)** Values of cross-entropy criteria (CEC) estimated for each putative number of genetic clusters (K). The best CEC value is displayed for each putative K value. **(b)** Group assignment at K=5. **(c)** Group assignment at K=6. **(d)** Group assignment at K=8.

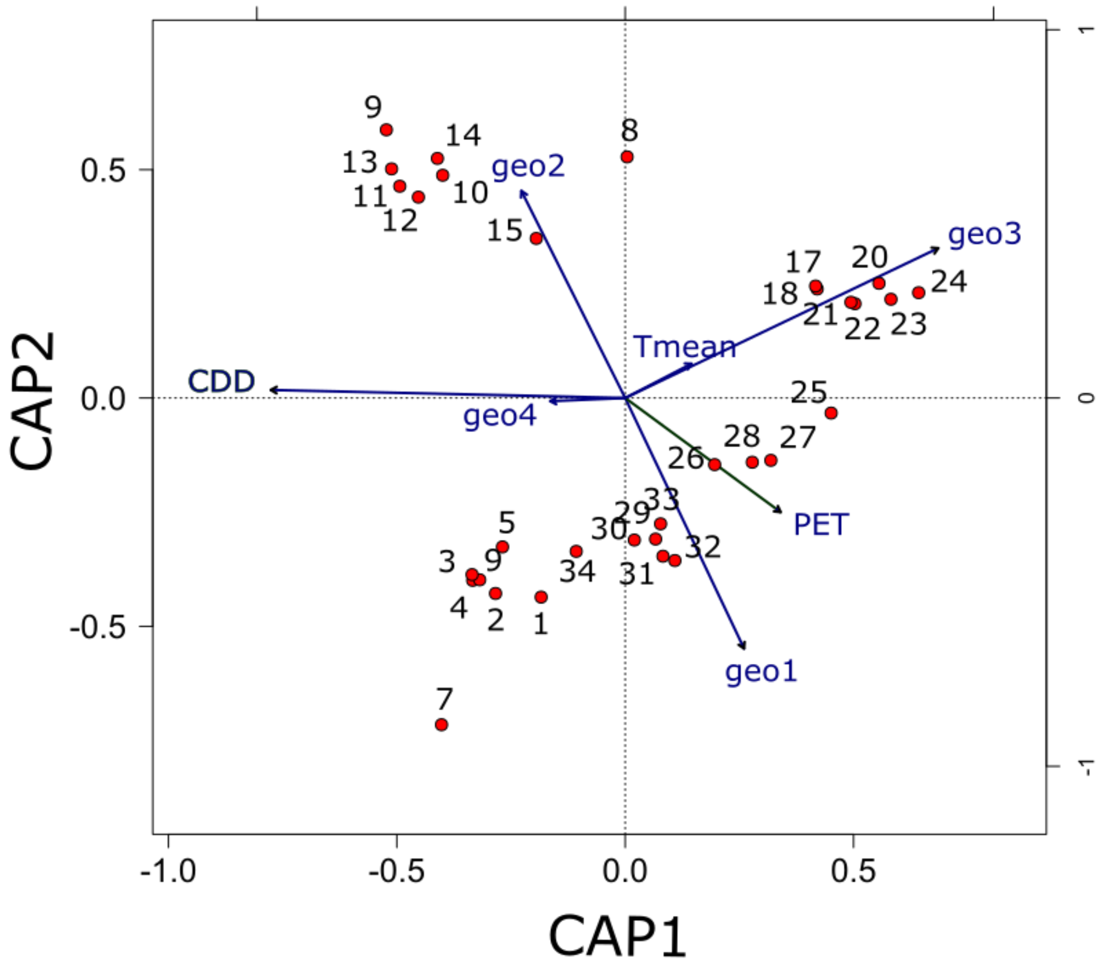

Figure S4. Biplot of RDA analysis. Information on source populations is given in table 1. Eigenvalues for constrained axes: CAP1=34.37%; CAP2=10.09%. Tmean: average daily temperature; CDD: mean number of consecutive dry days; PET: average monthly potential evapotranspiration; geo1, geo2, geo3: uncorrelated descriptors of geographic distances

Table S1. Species sampled for DArTseq experiment

Species	Origin	Acc. Code*	Biogeographic group
C. mauritiana	Reunion Island	1 to 326	Mascarenes
C. mauritiana	Mauritius	RM-CF-00599	Mascarenes
C. macrocarpa	Mauritius	RM-CF-00615	Mascarenes
C. bernardiniana	Mauritius	RM-CF-00638	Mascarenes
C. bernardiniana	Mauritius	RM-CF-00655	Mascarenes
C. bernardiniana	Mauritius	MPL-B	Mascarenes
C. myrtifolia	Mauritius	MPL-M	Mascarenes
C. myrtifolia	Mauritius	RM-CF-00662	Mascarenes
C. myrtifolia	Mauritius	RM-CF-00658	Mascarenes
C. bertrandii	Madagascar	RM-CF-00515	Madagascar
C. perrieri	Madagascar	RM-CF-00513	Madagascar
C. tsirananae	Madagascar	RM-CF-00514	Madagascar
C. dolichophylla	Madagascar	MPL-D	Madagascar
C. sessiliflora	Kenya	RM-CF-00487	East Africa
C. pseudozanguebariae	Kenya	RM-CF-00471	East Africa
C. eugenioides	Kenya	RM-CF-00422	East Africa
C. canephora	D.R. Congo	MPL-HD200-94	West and central Africa
C. liberica	Central African R.	RM-CF-00207	West and central Africa

* Accession numbers of either the IRD *Coffea* field genebank (RM-CF, www.intertrop.antilles.inra.fr/Portail/) or the greenhouse collection (MPL) are given.

Table S2. Pearson's correlation coefficients calculated between the 12 climatic variables, elevation, longitude and latitude. Pearson's correlation coefficients are below the diagonal and p-values calculated according to Holm's method are above.

	Tmin	Tmean	Tmax	Trange	Pmin	Pmean	Pmax	RCI	CDD	DSI	PET	WBmin	Elevation	Longitude	Latitude
Tmin		<0.001	<0.001	0.03	1	1	1	1	1	1	0.005	1	<0.001	1	1
Tmean	0.98		<0.001	0.85	1	1	1	1	1	1	0.01	1	<0.001	1	1
Tmax	0.82	0.91		1	1	1	1	1	1	1	0.86	1	<0.001	1	1
Trange	-0.59	-0.43	-0.02		0.06	0.002	0.14	1	0.001	<0.001	0.14	1	0.75	0.61	<0.001
Pmin	0.24	0.17	-0.1	-0.56		<0.001	<0.001	<0.001	<0.001	<0.001	0.24	<0.001	1	1	<0.001
Pmean	0.29	0.21	-0.12	-0.67	0.92		<0.001	<0.001	<0.001	<0.001	0.45	0.03	1	1	<0.001
Pmax	0.19	0.13	-0.14	-0.53	0.89	0.97		<0.001	<0.001	<0.001	1	0.01	1	1	<0.001
RCI	0.01	-0.02	-0.16	-0.25	0.85	0.79	0.88		<0.001	<0.001	1	<0.001	1	1	0.12
CDD	-0.29	-0.2	0.12	0.68	-0.97	-0.95	-0.89	-0.76		<0.001	0.08	0.01	1	1	<0.001
DSI	-0.41	-0.31	0.02	0.74	-0.92	-0.95	-0.89	-0.71	0.95		0.23	0.03	1	1	<0.001
PET	0.65	0.63	0.43	-0.53	0.5	0.47	0.41	0.28	-0.55	-0.5		1	0.02	1	0.03
WBmin	-0.24	-0.3	-0.41	-0.15	0.71	0.6	0.63	0.74	-0.62	-0.59	-0.24		1	1	1
Elevation	-0.98	-0.99	-0.89	0.44	-0.2	-0.22	-0.14	-0.01	0.22	0.34	-0.6	0.24		1	1
Longitude	-0.18	-0.13	0.1	0.45	-0.1	-0.32	-0.22	0.09	0.22	0.23	-0.31	0.28	0.14		0.02
Latitude	0.39	0.31	-0.04	-0.75	0.8	0.89	0.8	0.54	-0.88	-0.87	0.59	0.34	-0.33	-0.6	

Table S3. Orthodromic distances calculated between pairs of source populations

1	0																															
2	9,000	0																														
3	6,555	3,285	0																													
4	9,600	2,415	3,060	0																												
5	16,710	9,945	10,455	7,860	0																											
6	17,520	9,630	11,010	8,055	2,835	0																										
7	16,890	9,000	12,270	10,515	14,115	11,955	0																									
8	26,430	18,435	21,705	19,620	20,805	18,060	9,585	0																								
9	29,190	21,135	24,420	22,245	22,980	20,175	12,330	2,760	0																							
10	35,850	27,510	30,750	28,365	27,810	24,975	18,975	9,585	6,900	0																						
11	35,640	27,120	30,330	27,825	26,805	23,985	18,825	9,780	7,290	1,815	0																					
12	34,950	26,265	29,430	26,820	25,320	22,530	18,315	9,855	7,710	3,840	2,070	0																				
13	39,720	31,275	34,500	32,025	30,945	28,140	22,860	13,545	10,860	3,975	4,215	5,685	0																			
14	40,275	32,145	35,400	33,105	32,745	29,910	23,400	13,845	11,085	4,950	6,210	8,130	3,315	0																		
15	35,160	28,485	31,680	30,120	32,355	29,655	19,605	11,655	10,140	11,040	12,750	14,505	13,260	10,785	0																	
17	30,810	25,890	28,770	27,975	32,160	29,805	18,120	14,130	14,280	18,210	19,635	20,985	21,150	19,050	8,460	0																
18	29,610	25,575	28,230	27,780	32,655	30,465	18,555	16,170	16,725	21,180	22,530	23,775	24,255	22,230	11,685	3,225	0															
20	19,755	18,180	20,040	20,595	27,240	25,770	14,745	18,525	20,520	26,880	27,675	28,200	30,645	29,565	20,535	13,305	11,010	0														
21	20,490	20,010	21,570	22,410	29,355	28,020	17,295	21,195	23,130	29,415	30,255	30,825	33,135	31,935	22,575	14,880	12,210	2,715	0													
22	20,115	21,585	22,530	23,925	31,365	30,375	20,550	25,440	27,480	33,870	34,665	35,160	37,620	36,465	27,060	19,215	16,380	6,990	4,530	0												
23	20,940	23,430	24,060	25,710	33,315	32,490	23,100	28,260	30,330	36,705	37,515	37,995	40,455	39,270	29,775	21,795	18,870	9,840	7,350	2,850	0											
24	13,140	13,515	14,565	15,855	23,325	22,455	13,695	20,655	23,130	29,955	30,390	30,465	33,900	33,405	25,650	19,410	17,520	6,810	7,380	8,085	10,050	0										
25	21,795	30,615	28,335	31,380	38,115	39,240	37,080	45,930	48,600	55,500	55,650	55,335	59,460	59,340	51,870	45,135	42,720	31,830	30,570	26,895	25,170	26,250	0									
26	18,120	27,090	24,570	27,630	34,050	35,325	34,155	43,305	46,020	52,890	52,905	52,455	56,835	56,925	50,070	43,815	41,625	30,630	29,730	26,535	25,230	24,510	4,605	0								
27	17,055	25,965	23,565	26,625	33,255	34,425	32,790	41,865	44,565	51,450	51,510	51,090	55,395	55,440	48,450	42,120	39,915	28,935	27,990	24,780	23,490	22,860	4,890	1,755	0							
28	14,430	23,310	20,955	24,000	30,735	31,845	30,165	39,270	41,985	48,855	48,885	48,465	52,800	52,890	46,050	39,885	37,770	26,775	25,995	23,025	21,960	20,550	7,410	4,050	2,670	0						
29	10,065	19,035	16,530	19,590	26,205	27,360	26,325	35,640	38,385	45,195	45,135	44,610	49,125	49,395	43,125	37,500	35,655	24,765	24,405	22,125	21,615	18,150	11,925	8,070	7,080	4,545	0					
30	9,435	18,420	15,765	18,810	25,185	26,445	26,055	35,460	38,220	44,985	44,865	44,265	48,900	49,260	43,290	37,905	36,165	25,365	25,155	23,085	22,710	18,645	13,005	8,895	8,130	5,760	1,545	0				
31	7,590	16,575	13,920	16,965	23,400	24,630	24,285	33,720	36,480	43,230	43,080	42,465	47,130	47,535	41,730	36,540	34,875	24,195	24,150	22,365	22,215	17,415	14,730	10,710	9,870	7,380	2,865	1,860	0			
32	6,990	15,975	13,335	16,380	22,890	24,075	23,670	33,120	35,880	42,630	42,480	41,865	46,530	46,935	41,160	36,015	34,380	23,730	23,730	22,020	21,945	16,950	15,240	11,265	10,380	7,860	3,330	2,445	615	0		
33	7,260	16,185	13,440	16,470	22,770	24,060	24,075	33,555	36,315	43,035	42,855	42,195	46,920	47,370	41,745	36,690	35,100	24,495	24,540	22,875	22,800	17,700	15,405	11,295	10,530	8,100	3,615	2,415	840	870	0	
34	7,725	14,550	11,295	13,890	18,540	20,370	23,400	32,925	35,655	42,045	41,610	40,680	45,795	46,680	42,390	38,445	37,320	27,420	28,020	27,180	27,600	20,730	20,835	16,365	16,020	13,905	9,705	8,250	7,095	6,930	6,270	0
	1	2	3	4	5	6	7	8	9	10	11	12	13	14	15	17	18	20	21	22	23	24	25	26	27	28	29	30	31	32	33	34

Table S4. Pairwise F_{ST} of Weir and Cockerham calculated between 32 source populations of *C. mauritiana*. Population codes are given in Table 1.

W	1	0.00																															
W	2	0.08	0.00																														
W	3	0.07	0.03	0.00																													
W	4	0.08	0.04	0.03	0.00																												
W	5	0.07	0.07	0.05	0.05	0.00																											
W	6	0.09	0.08	0.07	0.06	0.05	0.00																										
W	7	0.17	0.13	0.13	0.12	0.14	0.15	0.00																									
NE	17	0.21	0.24	0.24	0.25	0.22	0.24	0.30	0.00																								
NE	18	0.21	0.25	0.24	0.24	0.22	0.24	0.31	0.03	0.00																							
E	20	0.25	0.28	0.28	0.28	0.26	0.27	0.33	0.07	0.08	0.00																						
E	21	0.23	0.26	0.26	0.26	0.24	0.25	0.31	0.05	0.06	0.03	0.00																					
E	22	0.23	0.26	0.26	0.26	0.24	0.25	0.32	0.06	0.06	0.05	0.03	0.00																				
E	23	0.25	0.28	0.28	0.28	0.26	0.28	0.34	0.08	0.08	0.07	0.04	0.02	0.00																			
E	24	0.27	0.30	0.30	0.30	0.28	0.30	0.36	0.10	0.11	0.08	0.06	0.06	0.07	0.00																		
C	8	0.17	0.18	0.17	0.18	0.16	0.18	0.24	0.13	0.13	0.16	0.15	0.15	0.17	0.20	0.00																	
NW	9	0.20	0.19	0.17	0.18	0.18	0.19	0.26	0.29	0.29	0.32	0.31	0.31	0.33	0.35	0.15	0.00																
NW	10	0.17	0.16	0.15	0.15	0.15	0.17	0.24	0.25	0.25	0.29	0.27	0.27	0.29	0.31	0.14	0.09	0.00															
NW	11	0.17	0.16	0.14	0.15	0.14	0.17	0.23	0.27	0.27	0.31	0.29	0.29	0.31	0.34	0.14	0.05	0.05	0.00														
NW	12	0.17	0.16	0.14	0.15	0.14	0.17	0.23	0.26	0.26	0.30	0.28	0.28	0.30	0.32	0.15	0.08	0.07	0.04	0.00													
NW	13	0.18	0.17	0.16	0.16	0.15	0.18	0.24	0.28	0.28	0.32	0.30	0.30	0.32	0.34	0.16	0.09	0.07	0.04	0.06	0.00												
NW	14	0.18	0.16	0.15	0.16	0.15	0.17	0.24	0.25	0.26	0.29	0.27	0.27	0.29	0.32	0.14	0.10	0.06	0.05	0.07	0.04	0.00											
NW	15	0.14	0.15	0.13	0.14	0.13	0.15	0.23	0.19	0.19	0.23	0.21	0.21	0.23	0.26	0.12	0.14	0.10	0.11	0.11	0.10	0.09	0.00										
SW	29	0.10	0.14	0.13	0.13	0.11	0.14	0.21	0.15	0.15	0.18	0.16	0.16	0.18	0.20	0.15	0.22	0.19	0.20	0.20	0.21	0.20	0.15	0.00									
SW	30	0.09	0.13	0.12	0.12	0.10	0.13	0.21	0.16	0.16	0.19	0.17	0.17	0.19	0.22	0.15	0.22	0.19	0.19	0.19	0.20	0.19	0.14	0.03	0.00								
SW	31	0.11	0.16	0.14	0.15	0.13	0.15	0.23	0.16	0.16	0.19	0.17	0.17	0.18	0.21	0.16	0.24	0.20	0.21	0.21	0.23	0.21	0.16	0.04	0.04	0.00							
SW	32	0.10	0.16	0.15	0.15	0.13	0.15	0.24	0.15	0.15	0.18	0.16	0.16	0.18	0.20	0.15	0.24	0.21	0.22	0.21	0.24	0.22	0.16	0.04	0.04	0.04	0.00						
SW	33	0.09	0.14	0.13	0.13	0.11	0.13	0.21	0.14	0.14	0.17	0.15	0.15	0.17	0.19	0.14	0.22	0.19	0.20	0.19	0.21	0.20	0.14	0.03	0.03	0.02	0.01	0.00					
SW	34	0.08	0.11	0.09	0.10	0.07	0.11	0.19	0.19	0.19	0.23	0.21	0.20	0.23	0.25	0.16	0.20	0.17	0.17	0.17	0.18	0.17	0.14	0.07	0.07	0.08	0.08	0.06	0.00				
SE/SW	26	0.14	0.18	0.17	0.17	0.15	0.17	0.25	0.13	0.12	0.15	0.13	0.12	0.14	0.17	0.15	0.25	0.21	0.22	0.22	0.23	0.21	0.16	0.08	0.08	0.09	0.09	0.07	0.11	0.00			
SE	25	0.21	0.25	0.24	0.25	0.23	0.24	0.31	0.10	0.11	0.12	0.09	0.08	0.08	0.12	0.18	0.31	0.27	0.29	0.28	0.30	0.27	0.21	0.15	0.15	0.15	0.15	0.13	0.19	0.09	0.00		
SE	27	0.17	0.21	0.21	0.21	0.19	0.21	0.28	0.12	0.13	0.14	0.11	0.11	0.12	0.15	0.17	0.28	0.24	0.26	0.25	0.27	0.24	0.19	0.09	0.11	0.11	0.11	0.09	0.14	0.06	0.09	0.00	
SE	28	0.15	0.19	0.18	0.19	0.17	0.18	0.26	0.10	0.10	0.12	0.10	0.09	0.10	0.13	0.14	0.26	0.22	0.24	0.23	0.25	0.23	0.17	0.07	0.09	0.08	0.08	0.07	0.12	0.05	0.07	0.04	0.00
		1	2	3	4	5	6	7	17	18	20	21	22	23	24	8	9	10	11	12	13	14	15	29	30	31	32	33	34	26	25	27	28
		W	W	W	W	W	W	W	NE	NE	E	E	E	E	E	C	NW	NW	NW	NW	NW	NW	NW	SW	SW	SW	SW	SW	SW	SE/ SW	SE	SE	SE

Table S5. Results from *f3-statistic* tests among the seven clusters defined by sNMF analysis. Only negative Z-score are shown. According to Reich *et al*. (2009), results were considered as significant when Z-score < -2. Admixt population = population tested for admixture between parent 1 and 2.

Admixt population	Parent 1	Parent 2	f3-statistic	s.e.	Z-score
SW	W	NE	-0.00109	0.000615	-1.77402
SW	W	SE	-0.00132	0.0004	**-3.28951**
SW	NW	SE	-7.35E-05	0.000497	-0.14774
SW	W	E	-0.00196	0.000659	**-2.98648**
SE	W	E	-0.00127	0.000599	**-2.12336**
SE	E	SW	-0.00063	0.000414	-1.51478
C	NE	NW	-0.00165	0.000845	-1.94858
C	NW	E	-0.00164	0.000948	-1.73423
